# Lithium Improves Survival of PC12 Pheochromocytoma Cells in High-Density Cultures and after Exposure to Toxic Compounds

**DOI:** 10.1155/2014/135908

**Published:** 2014-01-20

**Authors:** Cinzia Fabrizi, Stefania De Vito, Francesca Somma, Elena Pompili, Angela Catizone, Stefano Leone, Paola Lenzi, Francesco Fornai, Lorenzo Fumagalli

**Affiliations:** ^1^Department of Anatomy, Histology, Forensic Medicine and Orthopedics, Sapienza University, Via A. Borelli 50, 00161 Rome, Italy; ^2^Department of Science, Roma Tre University, Rome, Italy; ^3^Department of Human Morphology and Applied Biology, University of Pisa, Pisa, Italy; ^4^I.R.C.C.S. Neuromed, Pozzilli, Italy

## Abstract

Autophagy is an evolutionary conserved mechanism that allows for the degradation of long-lived proteins and entire organelles which are driven to lysosomes for digestion. Different kinds of stressful conditions such as starvation are able to induce autophagy. Lithium and rapamycin are potent autophagy inducers with different molecular targets. Lithium stimulates autophagy by decreasing the intracellular myo-inositol-1,4,5-triphosphate levels, while rapamycin acts through the inhibition of the mammalian target of rapamycin (mTOR). The correlation between autophagy and cell death is still a matter of debate especially in transformed cells. In fact, the execution of autophagy can protect cells from death by promptly removing damaged organelles such as mitochondria. Nevertheless, an excessive use of the autophagic machinery can drive cells to death via a sort of self-cannibalism. Our data show that lithium (used within its therapeutic window) stimulates the overgrowth of the rat Pheochromocytoma cell line PC12. Besides, lithium and rapamycin protect PC12 cells from toxic compounds such as thapsigargin and trimethyltin. Taken together these data indicate that pharmacological activation of autophagy allows for the survival of Pheochromocytoma cells in stressful conditions such as high-density cultures and exposure to toxins.

## 1. Introduction

Pheochromocytoma is a rare neuroendocrine tumour derived from chromaffin cells of the adrenal gland. Surgical resection of the tumour is the treatment of choice and usually results in cure of the hypertension related to the excessive release of catecholamines. Approximately, 17% of these tumours are malignant and treatment for metastatic disease includes surgical resection and chemotherapy with nonspecific agents which indiscriminately target dividing cells [1].

A previous report indicates a possible novel strategy for treatment of Pheochromocytomas and paragangliomas showing that lithium determines a net reduction of the growth in culture of the rat Pheochromocytoma cell line PC12 [2].

Lithium is already therapeutically widely used as a mood stabilizer in the treatment of bipolar disorders and in human patients levels of lithium in the serum are kept in the range of 0.4–1.2 mM [3]. Due to its diverse molecular targets, the action of lithium may be complex and the interpretation of its effects in biological systems is often controversial.

In fact, lithium is a monovalent cation with different cellular targets depending on its concentration. At 0.5–1 mM it acts mainly as an inhibitor of inositol monophosphatase (IMPase) (Ki 0.8 mM) leading to free inositol depletion and activating autophagy [4]. Autophagy is a catabolic pathway which delivers cellular components to lysosomes for digestion. First step is the engulfment of cytoplasmic material or entire organelles in autophagosomes which later on fuse with lysosomes to form autophagolysosomes. LC3 (microtubule-associated protein light chain 3) is localized in autophagosome membrane and is a widely applied marker for autophagy [5].

Conversely, when used at high doses lithium inhibits the glycogen synthase kinase-3 (GSK3) (Ki 1.5–2 mM) and reduces cellular proliferation and autophagy [6]. Two paralogs of GSK3 exist (GSK3*α*and GSK3*β*) usually referred to as isoforms because of their similar sequences and functions although they are derived from different genes [7]. These ubiquitously expressed serine/threonine kinases modulate a large number of cellular functions and their activity is inhibited by the phosphorylation of serine-21 in GSK3*α* and serine-9 in GSK3*β*.

Our data show that lithium if used in the range which corresponds to its therapeutic window (0.5–1 mM) favors the proliferation and survival of the rat Pheochromocytoma cell line PC12. Consistently with an activation of the autophagic pathway, 0.5 mM lithium induces the appearance of many autophagic vacuoles whereas the phosphorylation/inactivation of GSK3*α*/*β* was observed only at a higher lithium concentration (2 mM).

## 2. Materials and Methods

### 2.1. Cell Cultures and Treatments

PC12 rat pheochromocytoma cells (ECACC) were cultured in RPMI1640 and DMEM/F12 (1 : 1) supplemented with 5% foetal bovine serum, 10% horse serum, and 1% penicillin-streptomycin. Cells were incubated at 37°C in a humidified 5% CO_2_ atmosphere and medium was changed every three days.

PC12 cells were plated onto poly-L-ornithine-treated glass coverslips in 24 w plates (for fluorescence microscopy) or directly in 6-well or 96-well plates (for western blot analysis and assessment of neurotoxicity, resp.). PC12 cells were treated with LiCl (0.5, 1, 2 mM; Sigma) alone or in combination with thapsigargin (100 nM) or trimethyltin (TMT) (10 *μ*M; Heraeus, Karlsruhe, Germany). As for lithium treatment, rapamycin (400 nM; Sigma) was used alone or with TMT (1, 5, 10 *μ*M).

### 2.2. Proliferation Curve

PC12 cells were plated onto T25 culture flasks (5 × 10^5^ cells/mL) in 5 mL growth medium without antibiotics. Every 24 hours cells were resuspended in phosphate buffer and stained with Trypan blue. After 3 days of culture further 5 mL of growth medium was added. Viable cells were counted in triplicate by a hemocytometer.

### 2.3. Assessment of Cell Death

The DNA fragmentation of the apoptotic PC12 cells was detected using the terminal deoxynucleotidyl transferase-mediated biotinylated UTP nick end labeling (TUNEL) kit (In situ Cell Death Detection Kit, Roche). The cells were cultured on coverslips for 24 hours and at the end of the drug treatment fixed in 4% paraformaldehyde in phosphate buffer (pH 7.4) at room temperature for 15 min and then incubated with a permeabilizing solution (0.1% Triton X-100) for 10 min at 4°C. The cells were incubated with the TUNEL reaction mixture for 60 min at 37°C and visualized by inverted fluorescence microscopy (Eclipse E600, Nikon Instruments SpA, Italy). TUNEL-positive nuclei were counted in ten nonoverlapping fields per coverslip and then converted to percentage by comparing TUNEL-positive counts with the total cell nuclei as determined by DAPI (4′,6′-diamino-2-phenylindole) counterstaining.

Cell death was also evaluated by measuring the release of lactate dehydrogenase (LDH) in the culture medium by the Cytotoxicity Detection Kit (Roche, Mannheim, Germany) according to manufacturer's protocols.

### 2.4. Electron Microscopy

PC12 cells were treated with 0.5 mM lithium for 24 hours and then processed for electron microscopy. After washing with phosphate buffer 0.1 M (pH 7.4) samples were fixed in 0.1% glutaraldehyde and 2% paraformaldehyde in phosphate buffer 0.1 M (pH 7.4) for 1.30 hour at 4°C. After washing with phosphate buffer 0.1 M (pH 7.4) samples were postfixed in 1% OsO_4_ for 1 hour at 4°C and dehydrated in decreased series of ethanol and embedded in Epoxy resin. Ultrathin sections (40–50 nm) were cut at ultramicrotome. Sections were contrasted with uranyl acetate (saturated solution in methanol) and lead citrate and examined using a Jeol JEM SX 100 electron microscope (Jeol, Tokyo, Japan).

### 2.5. Detection of Autophagic Vacuoles by Immunofluorescence

PC12 cells were treated for 24 hours with 0.5 mM lithium and then fixed in 4% paraformaldehyde in phosphate buffer (pH 7.4) 1 hour at room temperature. After washings nonspecific antibody binding sites were blocked with 10% donkey serum (Sigma) and then cells were incubated for 1 hour at room temperature with a rabbit anti-LC3B antibody (Sigma) and binding of the primary antibody visualized by a secondary donkey Cy3-labelled-anti-rabbit-IgG (Jackson ImmunoResearch Laboratories). Immunolocalization was analyzed using a Leica confocal microscope (Laser Scanning TCS SP2) (Leica Microsystems, Wetzlar, Germany) equipped with Ar/ArKr and HeNe lasers. The images were scanned under a 40X oil immersion objective with electronic zoom of 2X and 4X. In order to perform a quantitative analysis, spatial series through the *z*-axis each composed of 9-10 optical sections with a step size of 2 *μ*m were performed and maximal amplitude of fluorescence was evaluated as described by [8] utilizing the Leica confocal software. No significant fluorescent signal was detected with the secondary antibody alone.

### 2.6. Western Blotting

Treated and untreated cells were lysed in RIPA buffer containing protease and phosphatase inhibitors (Protease Inhibitor Cocktail and Phosphatase Inhibitor Cocktail 2, Sigma). Samples were clarified by centrifugation at 1000 rpm for 5 min. Equivalent amount of protein (10 *μ*g) from each sample was electrophoretically resolved on 12.5% precast SDS-polyacrylamide gels (ExcelGel, GE Healthcare Biosciences) using horizontal apparatus (Pharmacia Biotech, Uppsala, Sweden). Then, separated proteins were electrotransferred onto nitrocellulose membranes (Schleicher & Schuell) by a semidry system (Novablot, Pharmacia Biotech). Membranes were blocked with 5% BSA in PBS and then were incubated (overnight at 4°C) with the following monoclonal antibodies: GSK3*α*/*β* (Sigma), phospho-GSK3*α*/*β* (Ser 21/9) (cell signalling). After extensive washing with PBS containing 0.1% tween-20 (TBST), blots were incubated with 1 : 2000 dilution of HRP-conjugated secondary antibody (Amersham Biosciences) for 1 hour at room temperature. Immunopositive bands were detected with a chemiluminescence's detection system (GE Healthcare Biosciences). To check for equal loading of the gel, membranes were stripped and reprobed with mouse anti-*β*-actin antibody (1 : 20000, Sigma). Densitometric analysis was performed with the Quantity One software (BioRad Laboratories).

### 2.7. Statistics

Statistical analyses were conducted using GraphPad Prism version 4.00 software. Data are expressed as averages ± SEM. Comparisons were analysed using one-way ANOVA with Bonferroni-corrected *t*-test. All experiments were performed at least three times.

## 3. Results

**Figure 1:**
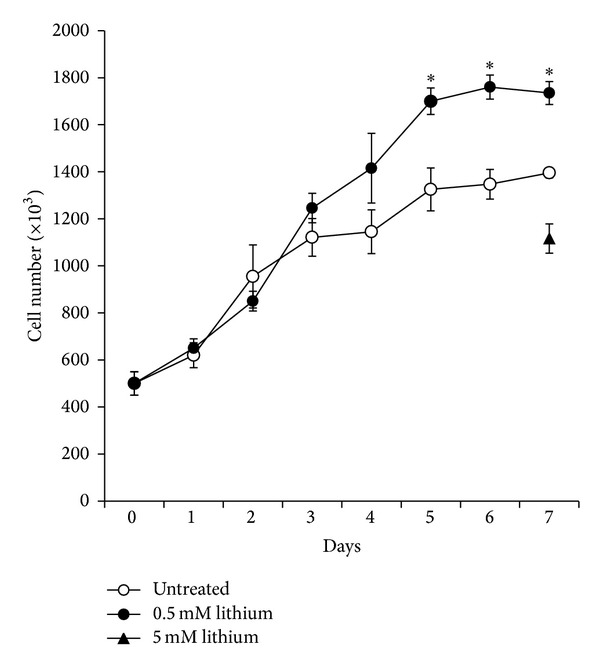
Proliferation curve of PC12 cells treated with lithium. Cells were treated with 0.5 mM lithium for 7 days and counted every day with the Trypan blue exclusion method. Counts obtained with 5 mM lithium at 7 days are shown as reference. Averages ± SEM of three independent experiments. ANOVA with Bonferroni's-corrected *t*-test. *P < 0.01 versus untreated control.

As aforementioned, a previous report indicates lithium as a possible novel treatment for Pheochromocytomas and paragangliomas [2]. Since lithium concentrations used in that study were beyond its potential therapeutic use, we decided to check the effect of lithium administration at the concentration measured in sera of lithium-treated patients. Similarly to Kappes and colleagues [2], we tested lithium administration in the rat Pheochromocytoma cell line PC12 which is widely used for toxicity studies. We noticed that after 5 days of culture 0.5 mM lithium increases PC12 cell number with respect to the untreated control, with this effect remaining stable up to 7 days of culture (Figure 1). A similar proliferation curve was obtained doubling the concentration of lithium to 1 mM while when PC12 cells were seeded at low density (2 × 10^5^/mL) neither 0.5 mM nor 1 mM lithium modified cell number with respect to control (not shown). Interestingly, no significant differences between treated and untreated cultures were observed before the proliferation curve reaches the plateau. BrdU incorporation analyses performed during the same period of time indicated a similar rate of DNA synthesis in untreated and lithium-treated cultures (not shown). Consistently with previously published results [2], lithium used at the concentration of 5 mM reduced cell number in PC12 cell cultures (Figure 1).

**Figure 2:**
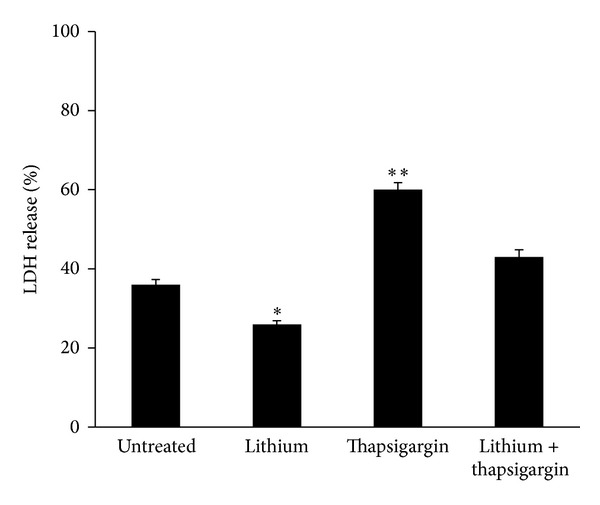
LDH release in the culture medium of PC12 cells treated for 24 hours with 100 nM thapsigargin alone or in combination with 0.5 mM lithium. Lithium treatment significantly reduces the thapsigargin-induced LDH release; a low but significant decrease in LDH release is observed in cultures treated with lithium alone with respect to the untreated control. Averages ± SEM of three independent experiments. ANOVA with Bonferroni's-corrected *t*-test. *P < 0.01, **P < 0.001 versus untreated control.

**Figure 3:**
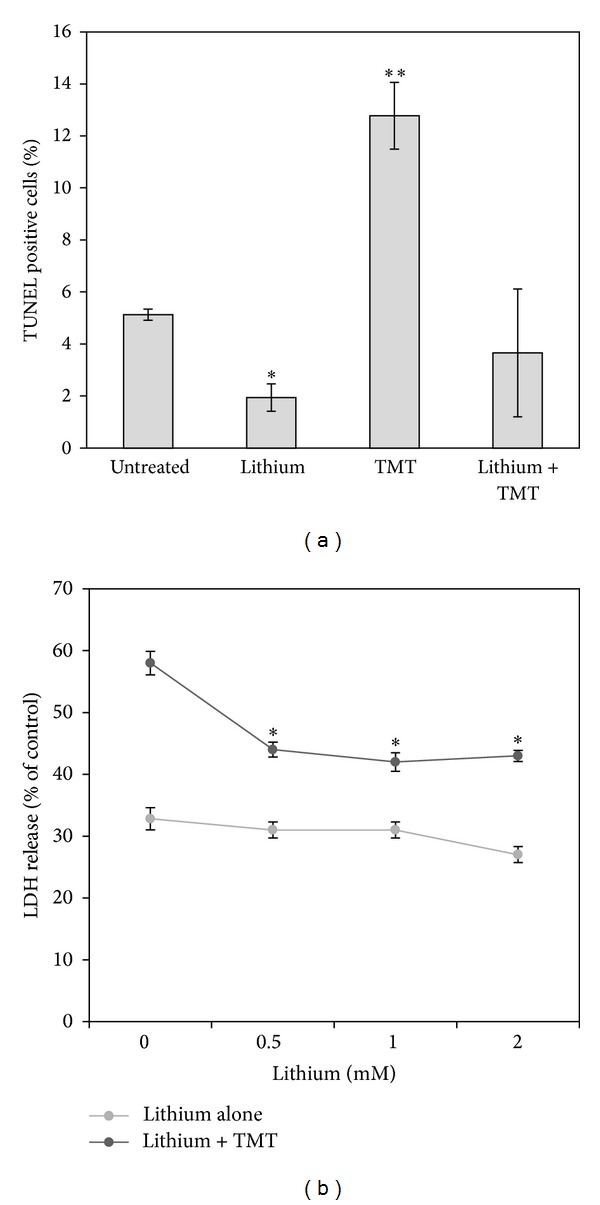
Detection of TUNEL-positive cells (a) and release of LDH (b) in the culture medium of PC12 cells treated for 24 and 48 hours, respectively, with 10 *μ*M TMT alone or in combination with 0.5 mM lithium. Lithium treatment completely reverses TMT-induced apoptosis (a); a significant inhibition of LDH release is observed in PC12 cells treated with doses of lithium ranging from 0.5 to 2 mM with respect to cultures treated with TMT alone. Averages ± SEM of three independent experiments. ANOVA with Bonferroni's-corrected *t*-test. *P < 0.01, **P < 0.001 versus untreated control.

In order to check if lithium at 0.5 mM was effective in inhibiting PC12 cell death, we administered this cation in combination with two different toxic molecules, thapsigargin and trimethyltin (TMT). As shown in Figure 2, lithium was able to almost completely rescue PC12 cells from thapsigargin-induced cell death. Similarly, lithium protected PC12 cells from TMT-induced apoptosis measured by TUNEL at 24 hours (Figure 3(a)). At 24 hours of TMT treatment PC12 cells seem to die mainly by apoptosis since the release of LDH in the culture medium remained similar to control (not shown). The release of LDH measured at 48 hours after TMT administration, which comprehends both necrosis and late apoptosis, was also reduced by lithium (Figure 3(b)).

Since lithium is known to be able to activate the autophagic pathway in different cellular systems, we checked the presence of autophagic vesicles in lithium-treated PC12 cells.

**Figure 4:**
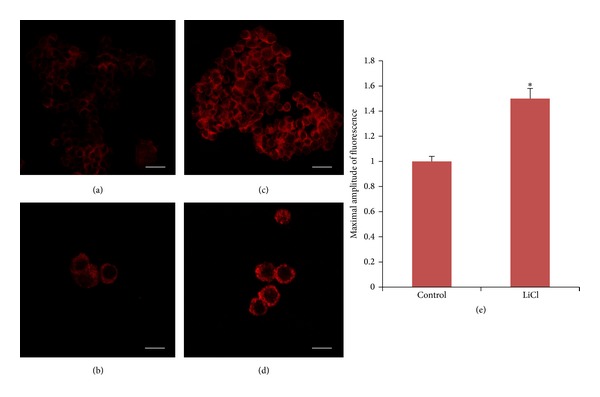
Confocal microscopy of LC3B distribution in PC12 cells after treatment for 24 hours with 0.5 mM lithium. Left panel: representative image of control ((a), (b)) and lithium-treated samples ((c), (d)). Scale bars = 20 *μ*m ((a), (c)); 40 *μ*m ((b), (d)). (e): quantitative analysis. The data represent the mean ± SE of maximal amplitude of fluorescence. *P < 0.001 versus control.

The expression of the autophagosome marker LC3B was evaluated by means of confocal microscopy in the control and lithium-treated samples. In lithium-treated PC12 the fluorescence intensity of LC3B was more evident and the mean value of the maximal amplitude of fluorescence was significantly increased (approximately 50%) with respect to the control (Figure 4).

**Figure 5:**
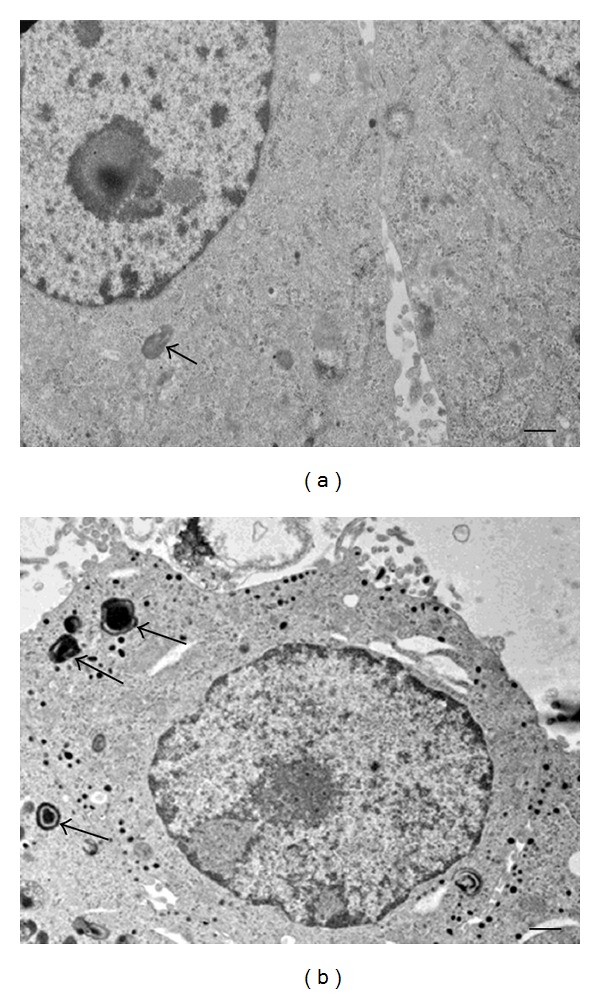
Ultrastructural analysis of lithium-treated PC12 cells. Membrane-delimited vacuoles in the cytoplasm with the characteristic features of autophagic vacuoles (arrows) are present in untreated cells (a) and more numerous in lithium-treated sample (b). Scale bars = (a): 1 *μ*m; (b): 0.4 *μ*m.

Many autophagic vesicles were also detected by electron microscopy in lithium-treated cultures (Figure 5).

**Figure 6:**
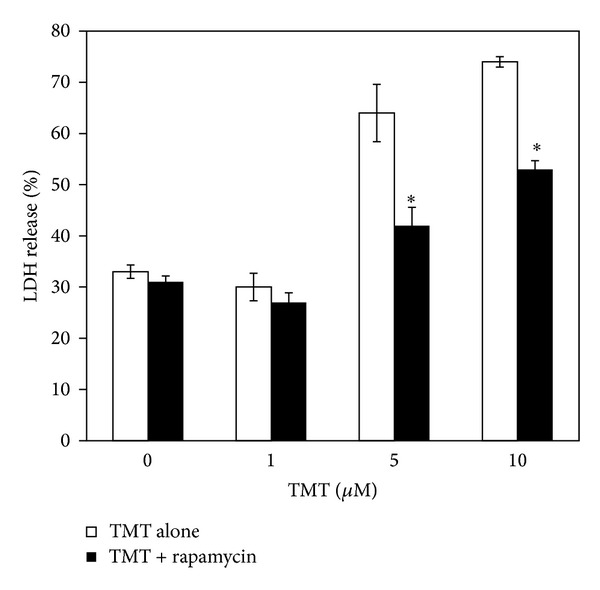
Rapamycin protects PC12 cultures from TMT toxicity. Rapamycin (400 nM) limits the release of LDH in the culture medium following 5–10 *μ*M TMT exposure and its effect lasts up to 72 hours. ANOVA with Bonferroni's-corrected *t*-test. *P < 0.01 versus TMT alone.

Besides, another known autophagy inducer such as rapamycin similarly to lithium was effective in reducing TMT neurotoxicity (Figure 6) confirming that a prompt activation of the autophagic machinery can protect PC12 from cell death.

**Figure 7:**
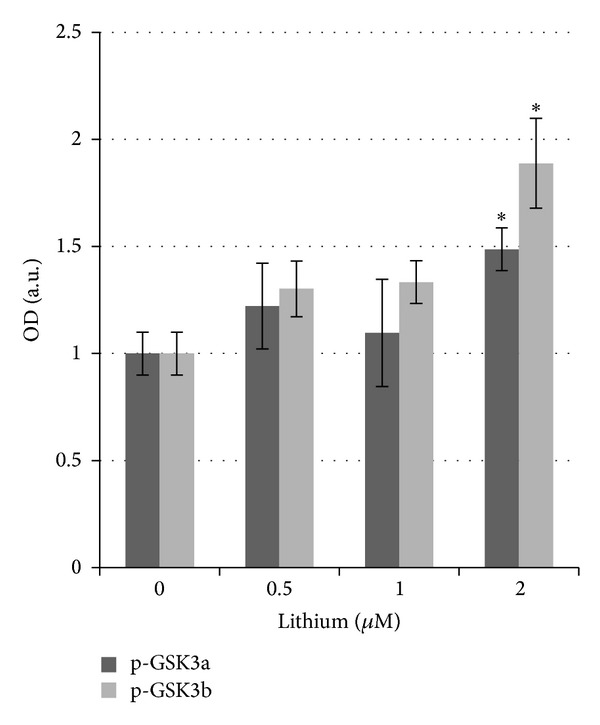
Analysis of the level of GSK3 phosphorylation in PC12 cells treated with lithium (0.5, 1, 2 mM) for 48 hours. Quantification data of western blot analysis of the phosphorylation of GSK3*α* Ser^21^ and GSK3*β* Ser^9^. Averages ± SEM of three independent experiments. ANOVA with Bonferroni's-corrected *t*-test. *P < 0.01 versus untreated cells.

We also checked in our cultures the phosphorylation of GSK3 which is a well-known lithium target. The phosphorylation/inactivation of GSK3 resulted to be unchanged after treatment with 0.5 mM lithium, while at 2 mM a statistically significant increase in the phosphorylation of both the GSK3*α* and GSK3*β* isoforms was measured with respect to the untreated control (Figure 7).

## 4. Discussion

How the autophagic pathway can be involved in the progression of cancer is still a matter of controversy. In fact, autophagy has been reported to participate in both tumour suppression and tumour maintenance (for a recent review see [9]). On the one hand, loss of genes which govern the autophagic machinery such as beclin1 is observed in various cancers [10]. On the other hand, autophagy by limiting oxidative stress and supplying metabolic substrates can promote tumour growth and maintenance [11, 12].

One way to reconcile these apparently conflicting results is to speculate that the correct execution of autophagy prevents the initial phase of tumourigenesis while later on in the progression of cancer the same autophagic machinery can be used by transformed cells to survive in adverse growth conditions which are present inside a solid tumour due to the limited blood supply.

In this connection, our data support this interpretation indicating autophagy as a prosurvival mechanism for tumour cells growing in a stressful environment, such as an overpopulated culture.

As mentioned before, lithium has been proposed for the treatment of Pheochromocytomas and paragangliomas [2]. These authors demonstrated that this cation can block the proliferation of PC12 cells in culture. The minimum dose of lithium showing this antiproliferative effect is 5 mM and thus far beyond its potential therapeutic use. In fact, lithium has a narrow therapeutic window and well-known adverse effects. Serum levels of lithium ranging from 0.4 to 1.2 mM are effective in the treatment of bipolar disorders and show minor side effects [3]. Conversely, prolonged exposures to serum levels of 2 mM lithium lead to renal and liver damage and permanent neurological impairment is observed with plasma levels above 2.5 mM [13].

When we used lithium in a range of concentration corresponding to 0.5–1 mM we observed a completely different result with respect to Kappes and colleagues [2]. In fact, PC12 cells in the presence of 0.5 mM lithium grow as the untreated control during the first 3 days of culture showing a similar exponential phase. Afterwards, in lithium-treated PC12 cells a higher cellular number was reached with respect to control at 5 days and maintained up to 7 days during the stationary phase. These results are not consistent with a direct mitogenic activity of lithium. In particular, lithium seemed to favor the growth of PC12 cells just in high-density cultures which likely grow in stressful conditions due to shortage of nutrients and accumulation of catabolites.

Besides, our data indicate that lithium at 0.5 mM exerts a neuroprotective action in PC12 cells treated with two toxic molecules (thapsigargin and TMT) which act with different mechanisms. Thapsigargin is a potent inhibitor of the Ca2+-ATPase in the endoplasmic reticulum [14] and our results are in agreement with a previous report which indicates that lithium can revert thapsigargin toxicity in a range of concentration from 0.5 to 4 mM [15]. TMT is another molecule highly toxic for PC12 cells determining a strong loss of the mitochondrial membrane potential correlated with an increased expression of bax/bcl-2 ratio [16]. TMT toxicity relates to the expression of stannin, a highly conserved protein mainly localized within mitochondria [17]. We show here that in PC12 cultures the addiction of lithium reduces the percentage of TUNEL-positive apoptotic nuclei completely rescuing PC12 from TMT toxicity. Lithium administration also limits the release of LDH in the culture medium which is observed during necrotic cell death and in the late stages of apoptosis. In agreement with data reported here, our previous results indicate that lithium (0.5–2 mM) protects hippocampal and cortical primary neurons from TMT-induced cell death [18]. Moreover, 1.2 mM lithium was also shown to reverse the effect of morphine on the mRNA expression of bax and bcl-2 in PC12 cells [19].

Lithium is a cation with a complex mechanism of action. Its main molecular targets are IMPase and GSK3. For the former the reported Ki is 0.8 mM [4] while for the latter it corresponds to 1.5–2 mM [6]. Thus, it is very likely that completely different biological effect of the same molecule (lithium) can be observed just changing its concentration. In this study we show the appearance of numerous autophagic vesicles in PC12 cells after treatment with 0.5 mM lithium while at this low concentration of lithium the phosphorylation/inactivation of GSK3 was not observed. Besides, lithium neuroprotection with respect to TMT toxicity can be mimicked by a known autophagy inducer such as rapamycin. Nevertheless, the neuroprotective effect of lithium observed in our culture system can also be related to an increased synthesis and release of trophic factors. In fact, long-term lithium chronic treatment has been demonstrated to enhance BDNF and NT-3 expression in vivo [20, 21].

We conclude that lithium when used at low doses (0.5 mM) can protect PC12 cells from toxic molecules and can also allow the overgrow of these cells in high-density cultures. Thus, caution should be taken for the clinical use of this cation in tumour bearing patients.
